# Gene editing in plants: assessing the variables through a simplified case study

**DOI:** 10.1007/s11103-020-00976-2

**Published:** 2020-02-10

**Authors:** Jay Shockey

**Affiliations:** Agricultural Research Service, Southern Regional Research Center, Commodity Utilization Research Unit, United States Department of Agriculture, 1100 Robert E. Lee Blvd., New Orleans, LA 70124 USA

**Keywords:** CRISPR, Single-guide RNA, Cas9, Hydroxy fatty acids, Gene editing, Arabidopsis

## Abstract

**Key message:**

Multiple variables that control the relative levels of successful heritable plant genome editing were addressed using simple case studies in *Arabidopsis thaliana*.

**Abstract:**

The recent advent of genome editing technologies (especially CRISPR, Clustered Regularly Interspaced Short Palindromic Repeats) has revolutionized various fields of scientific research. The process is much more specific than previous mutagenic processes and allows for targeting of nearly any gene of interest for the creation of loss-of-function mutations and many other types of editing, including gene-replacement and gene activation. However, not all CRISPR construct designs are successful, due to several factors, including differences in the strength and cell- or tissue-type specificity of the regulatory elements used to express the Cas9 (CRISPR Associated protein 9) DNA nuclease and single guide RNA components, and differences in the relative editing efficiency at different target areas within a given gene. Here we compare the levels of editing created in *Arabidopsis thaliana* by CRISPR constructs containing either different promoters, or altered target sites with varied levels of guanine–cytosine base content. Additionally, nuclease activity at sites targeted by imperfectly matched single guide RNAs was observed, suggesting that while the primary goal of most CRISPR construct designs is to achieve rapid, robust, heritable gene editing, the formation of unintended mutations at other genomic loci must be carefully monitored.

**Electronic supplementary material:**

The online version of this article (10.1007/s11103-020-00976-2) contains supplementary material, which is available to authorized users.

## Introduction

Deciphering gene function is often a long and difficult process. For decades, the best hope for beginning such a task was the availability of a workable biological system containing a mutation in the gene of interest. Such mutations often create changes in the chemistry, anatomy, or physiology of the organism that help to reveal the gene’s purpose. Certain model systems, such as *Escherichia coli*, baker’s yeast, *Chlamydomonas reinhardtii*, *Arabidopsis*, and maize have proven amenable to production and screening of large mutant populations, which were created through treatment with chemical or biological mutagens, such as UV light, ethylmethane sulfonate (EMS) or foreign DNA (Haughn and Somerville 1986; Shrager et al. 2003). These techniques were powerful, but each treatment generated differing numbers of mutations that were randomly distributed throughout the genome, and often did not generate mutations in every gene. The random nature of the mutagens, combined with the lack of complete mutational coverage (McCourt and Benning 2010; O’Malley and Ecker 2010) often limited the full application of such techniques to small organisms with a short life cycle, and/or those compatible with simple, high-throughput screens. Many agronomic plant species are not susceptible to some of the mutagenic treatments or are logistically incompatible with screening large numbers of randomly mutated lines, thus hampering the collective ability to study gene function in the most useful biological contexts.

Instead of screening whole populations for specific mutations with no guarantee of success, geneticists and biochemists fantasized for decades about the power to create targeted, specific mutations in the gene of choice. Fortunately, the advent of targeted genome editing technologies such as CRISPR-Cas9 recently unleashed the power to do just that. CRISPR genome editing was originally identified in *Streptococcus pyogenes* (Jinek et al. 2012) and has since been found in many other bacteria and archaea. Three CRISPR systems are known and all are thought to provide mechanisms for innate immunity against viruses, though each contains some unique characteristics. The seminal type II system CRISPR is a relatively simple two-component system containing a nuclease and a single guide RNA (sgRNA) that provides both the target DNA binding and Cas9-interacting domains (Jinek et al. 2012). The sgRNA contains a 20 nucleotide region that is complementary to a target region of the gene of interest. This 20 nucleotide region, called the protospacer, and a three base-pair domain adjacent to it (the protospacer adjacent motif, or PAM, defined by the consensus sequence 5′-NGG-3′) is all that is necessary to specify the sgRNA target site and to direct the nuclease. In the presence of the sgRNA, Cas9 cleaves the bound DNA, inducing a double-stranded break (DSB), which is repaired by the host. The repair process is often imperfect, leading to frameshift-inducing insertions and deletions, forming mutations in the gene of interest.

An ever-more comprehensive set of CRISPR-based genetic tools has been developed over the past few years, and the promise of making a specific mutation in a given gene in bacterial, yeast, plant, animal, or human cells and whole organisms has never been greater. Moving beyond simple gene knockouts, CRISPR technologies have been developed into a large and continuously expanding repertoire of sophisticated tools that can generate other, more complex types of genetic modifications. These include gene replacement (‘gene knock-in’) (Butler et al. 2016), transcriptional activators and repressors (Gilbert et al. 2013; Lowder et al. 2015; Piatek et al. 2015), and ‘base editing’, in which specific target site nucleotide bases are enzymatically interconverted, without the need for DNA strand breakage and repair, thus creating premature stop codons or changes to the amino acid sequence of the encoded protein of the target gene (Zong et al. 2017; Yan et al. 2018; Kumlehn et al. 2018).

All CRISPR experiments, regardless of the organism to be used, face limitations regarding laboratory space, materiel availability, cost, etc. Therefore, the goal of such experiments is to find samples or lines that contain homozygous mutations in the gene of interest, which are established as early in the genetic lineage as possible, are as specific to the gene of interest as possible, all while requiring screening as few initial independent transgenic events as possible.

Given the intellectual critical mass that has been focused on this technology, CRISPR-based gene editing has been applied with impressive effects over the past several years in numerous systems, including bacteria, algae, plants, animals, and human cells, with literally dozens of new publications appearing daily. Despite these impressive accomplishments, the full power of this technology likely has not been fully realized in nearly as many laboratories as possible, for multiple reasons. One is the increasingly overwhelming number of gene and plasmid choices available. Plasmid repositories such as Addgene (https://www.addgene.org/crispr/guide/#overview) and publicly available optimization tools from private laboratories (Lowder et al. 2015; https://chopchop.cbu.uib.no; Labun et al. 2016; https://cfans-pmorrell.oit.umn.edu/CRISPR_Multiplex/vector.php; Čermák et al. 2017) continue to do admirable and extremely important work in trying to provide both the resources for successful gene editing experiments and informed guidance for their use.

Multiple variables must be addressed, including which nuclease enzyme to choose (Cas9 being most common, but including others with different protospacer and PAM sequence requirements) (Steinert et al. 2015; Tang et al. 2017), and which promoters and terminators to use for optimal expression of the Cas nuclease and sgRNA genes. Perhaps most important is sgRNA design, which requires not only examination of which region of the gene of interest to target, but also the type of genetic module to use for production of the sgRNA. Most published studies describing targeted mutagenesis of specific genes may not provide a full reporting of the numbers and types of Cas/sgRNA variations that were tested, logically focusing only on the construct designs that were successful.

Several laboratories have realized the potential utility of user-friendly computer algorithms that could demystify the sgRNA design process (Lei et al. 2014; Labun et al. 2016, 2019; Liang et al. 2016). The persistent variability in specific targeting efficacy, as suggested by the number of publications that continue to address the topic, strongly suggests that sgRNA choice remains the critical variable, and that the goal of ‘fool-proof’ sgRNA design has not yet been achieved (“At the same time, even beautiful gRNAs fail for no clear reason.”—Lukas Dow, Weill Cornell Medicine, in Marx 2018).

Described herein are the results of our efforts to understand the precepts of design and testing of simple yet reasonably effective CRISPR-based plant gene editing plasmid constructs, using straightforward test cases. The results described here may help to simplify some of the decision-making required for setting up CRISPR technology for the first time, thus reaching laboratories that have not yet utilized gene editing techniques. We focused primarily on identification of optimal regulatory elements to drive *Cas9* and *sgRNA* expression leading to heritable mutations, and an analysis of *sgRNA* efficacy and specificity, through comparisons of protospacer sequence content and targeted *sgRNA* mutants.

## Results

### Some promoter types confer rapid gene-editing activity, but not heritable mutations

Key to any successful genetic engineering approach is the use of appropriate regulatory elements to appropriately control the timing, strength, and cell-type specificity of transgene expression. Most early plant CRISPR studies, and many current ones, have relied on two general categories of promoters: RNA polymerase III-specific promoters (such as U3 in monocot species, and U6 in dicots) (Lowder et al. 2015) for expression of the sgRNA molecules, and strong, ubiquitous promoters (often derived from viral or bacterial plant pathogens, Bevan 1984; Gleave 1992) to drive Cas9 expression. The RNA polIII class of promoters can only effectively be used for high-level production of small transcripts, making them a good choice for expression of the sgRNA component, which is typically ~ 100–200 nucleotides in length. However, Cas9 encodes for a large protein (~ 1400 amino acid residues), and therefore must be expressed using other promoters.

**Fig. 1:**
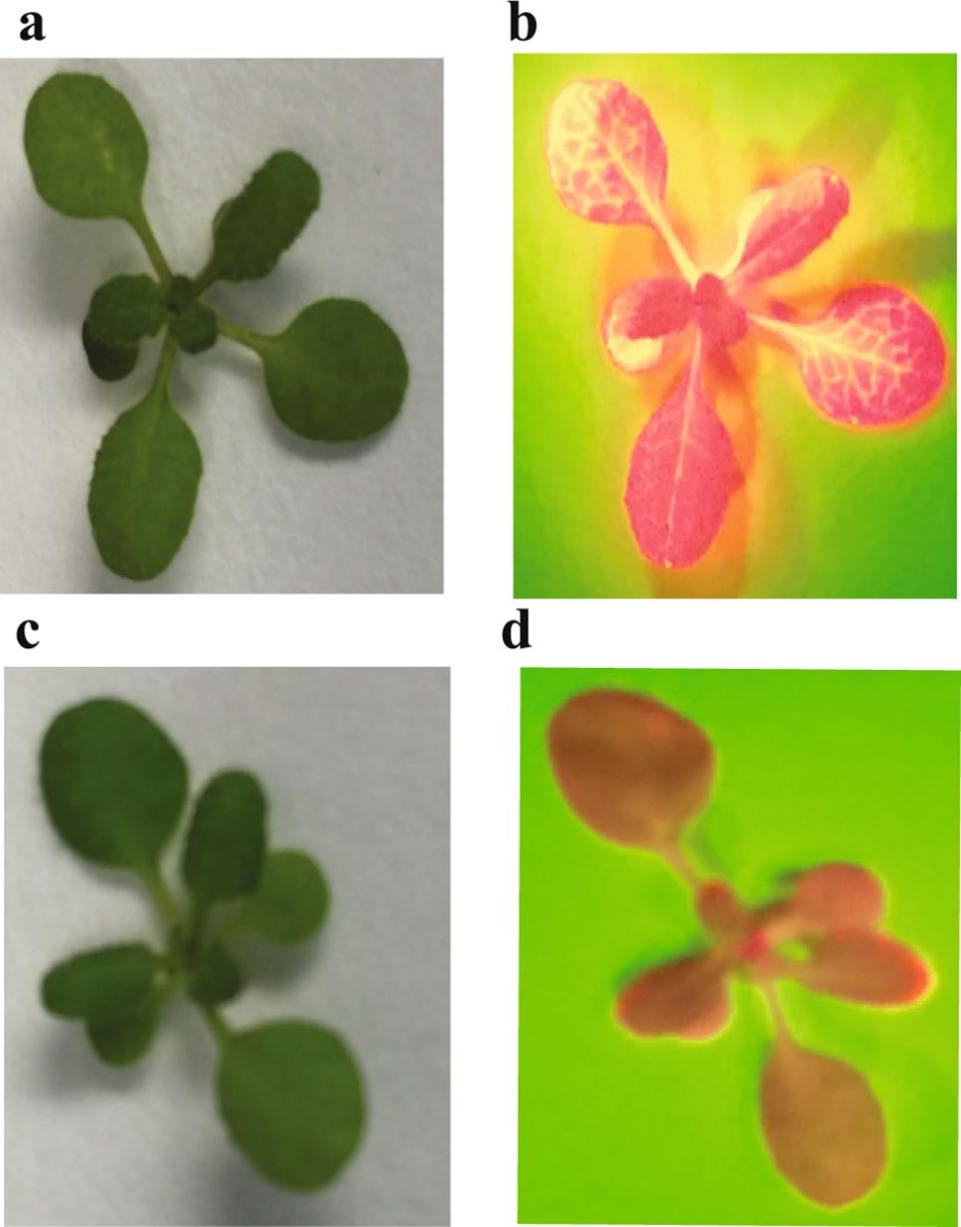
Visual inspection of 14-days old *Arabidopsis* E113 plants expressing different CRISPR constructs. Control plant expressing Cas9 alone visualized in normal light (**a**) or green light with a red filter (**b**) to visualize red fluorescence from the constitutively expressed *DsRed* gene. A representative plant expressing Cas9 and a sgRNA targeting *DsRed*, shown in normal light (**c**) or green light with a red filter (**d**)

Using this arrangement of genetic components, many previous plant CRISPR studies reported high levels of Cas9 activity in vegetative tissues of first generation transgenic plants (Feng et al. 2014; Shan et al. 2018). In the early stages of our work, we also observed similar results. The first test subject was *Arabidopsis thaliana* E113, a transgenic line engineered to produce ~ 7–8% α-eleostearic acid in its seeds (Dyer et al. 2002; Shockey et al. 2015). The marker for selection of transgenic E113 plants is the *DsRed* fluorescent protein gene. To test our first generation CRISPR/Cas9 constructs, we re-transformed E113 plants with *Agrobacterium tumefaciens* expressing three different binary plasmid constructs. Each contained the plant codon-optimized Cas9 gene, flanked by the *nos* promoter and *nos* terminator (Gleave 1992; Li et al. 2013). Constructs E642 and E640 also contained sgRNA components targeted to either of two different PAM sites located at nucleotide positions 341 and 422, respectively, in the 678 bp *DsRed* open reading frame. Plasmid construct E638 contained the *Cas9* gene alone, and was used as a negative control. Glufosinate-resistant seedlings were selected for each of the three constructs by growth on soil wetted with herbicide solution, then transferred to untreated soil after the first pair of true leaves had fully expanded. One week after transplanting, the vegetative tissues were viewed through a red filter, with green light illumination. Under these conditions, DsRed-based fluorescence was readily detectable in vegetative tissues of all control E638 plants (Fig. 1b). Vegetative tissue of ~ 67% of E642 plants (Fig. 1d) and ~ 20% of E640 plants (not shown) displayed significant loss of red fluorescence, demonstrating ample proof of concept that the Cas9 protein was expressed at reasonable levels and that active, self-spliced sgRNAs targeting *DsRed* were produced from the ribozyme-based construct designs (Gao and Zhao 2014). Consistent with previous studies, Cas9 activity and error-prone DNA repair lead to several types of insertion and deletion mutations in the *DsRed* gene in these plants, as shown in Supplementary Fig. 1.

### Other promoters promote better expression in germline cells

Cas9 fused to strong, constitutive promoters can produce some stably inherited mutations in some cases (Feng et al. 2014; Castel et al. 2019). But most often, the mutations generated by CRISPR constructs with this structure are somatic, and thus confined to vegetative tissues, as we observed in Fig. 1. However, as in other past studies (Feng et al. 2014; Shan et al. 2018), the high degree of somatic mutations associated with the loss of T_1_ vegetative tissue red fluorescence was not reliably transmitted through the germline of these plants. Sixty-four individual glufosinate-resistant T_1_ plants from each of the three constructs were grown to maturity and seeds harvested. The consistent bright fluorescence levels seen in all 64 samples from the parental E113 line transformed with the negative control E638 construct (lacking a *sgRNA* element, Supplementary Fig. 2) did not change appreciably in any of the 128 *DsRed sgRNA*-bearing E640 or E642 T_2_ seed samples that were analyzed (Supplementary Fig. 3). Some previous studies have demonstrated occasional heritable mutation production via use of strong constitutive promoters (Feng et al. 2014), but these results indicated that the combined use of *nos* and *CaMV35s* promoters for expression of *Cas9* and *sgRNA*, elements, respectively, was insufficient for frequent production of heritable mutations at phenotypically obvious scale.

The barrier to generation of heritable mutations at reasonable frequencies likely requires more appropriate timing of expression of the two CRISPR components in germline cells and reproductive tissues. Fortunately, some recent reports have described other promoters that are strongly expressed in egg cells, embryos, pollen, flowers, siliques, and other reproductive cell and tissue types; some of these promoters and other regulatory elements have shown promise in helping to achieve heritable Cas9-derived mutations (An et al. 1996; Wang et al. 2015; Yan et al. 2015; Zhang et al. 2016). However, with notable exceptions (Castel et al. 2019), few direct comparisons of these promoters exist, so accurate assessments of their relative efficacy and consistency may often still be hard to deduce from the literature. We therefore decided to directly compare several possible combinations of regulatory elements, using constructs containing ribozyme-based sgRNA and plant codon-optimized Cas9.

In Fig. 1, we sought to assess the strength of CRISPR activity by a phenotypic screen (inspection for loss of red fluorescence), the visual nature of which greatly reduces the time and effort needed to detect mutations occurring at meaningful levels. Some previous studies have discussed gradual generational increases in levels of Cas activity in transgenic plant lines, eventually leading to robust levels of mutant detection in T_3_, and later, generations (Morineau et al. 2017). The phenotype-based approach we chose may overlook some ‘slightly active’ designs that gradually generate mutations in T_1_ and T_2_ plants, which may lead to a high rate of false negatives. But we felt that the practical demands for detection of ‘early’ mutations at an easily detected scale outweighed these concerns. Given our interest in seed lipid metabolism, in the following experiments we developed a similarly rapid and facile system for detection of heritable mutations generated at a scale sufficient for alteration of total seed fatty acid composition. The CL37 line of *A. thaliana* produces unusual hydroxylated HFA in seed lipids, due to stable overexpression of the castor bean fatty acid hydroxylase gene *FAH12* (Lu et al. 2006). CL37 plants were transformed with a series of nine *A. tumefaciens* strains expressing a binary plasmid construct containing modules for Cas9 and an sgRNA targeting PAM position 333 of *FAH12*, each fused to a distinct combination of the *YAO* promoter (Yan et al. 2015), *AtACT8* promoter (An et al. 1996), *EC1.2p*-enhanced *EC1.1* promoter (Wang et al. 2015), or *AtUBQ10* promoter (Zhang et al. 2016). To avoid any bias towards selection of somatic mutations, at least 6–8 transgenic T_1_ seeds were selected randomly, and grown to maturity, but not analyzed in more detail. Segregating T_2_ seed samples were harvested from each parental T_1_ plant and total seed fatty acid composition was determined by separation and quantification of fatty acid methyl esters by gas chromatography (GC). Any significant changes to T_2_ seed HFA content, relative to parental CL37 controls, would represent *FAH12* mutations generated during the progression through the T_1_ generation and during T_2_ seed development, including those events that were heritably transmitted from the T_1_ mother plants to T_2_ seeds.

**Fig. 2:**
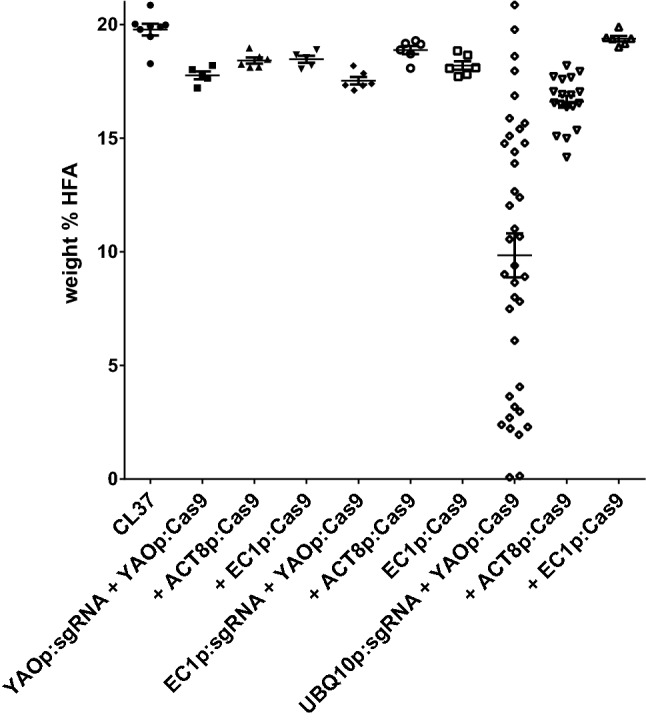
Testing the relative effectiveness of different promoters in dual sgRNA/Cas9 CRISPR plasmid designs. Shown here are HFA levels in segregating T_2_ seed samples derived from *Arabidopsis* CL37 plants transformed with CRISPR constructs containing different combinations of promoters fused to Cas9 and a sgRNA targeting the region proximal to nucleotide position 333 of the *RcFAH12* ORF. Randomly chosen red T_1_ seeds were sown on soil and grown to maturity, followed by seed harvest and GC analysis of total seed FAME composition. The x-axis lists the promoters fused to each sgRNA and Cas9 element, while the Y-axis represents the weight% of the HFAs present in each seed sample. Each data point represents the seeds from an individual transgenic T_1_ event. The bars in each data set represent the average and standard error of measurement. Unmodified CL37 plant seed samples are included as controls

**Fig. 3:**
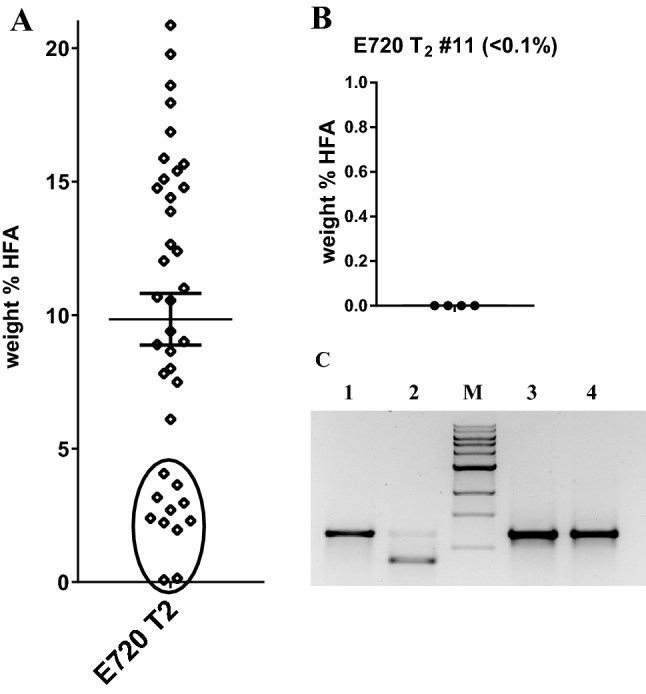
Assessment of heritability of mutations acquired in low-HFA CL37 lines transformed with hydroxylase gene editing constructs. **a** The distribution of HFA levels in segregating T_2_ seeds samples from lines expressing the *UBQ10p*:*sgRNA* + *YAOp:Cas9* binary construct (as shown in Fig. 2, also called construct E720). Lines containing strongest decreases in HFA compared to controls are circled. **b** HFA levels in T_3_ seeds produced from four brown (i.e. Cas9 T-DNA-free) seeds from line E720 T_2_ #11, which contained < 0.1% HFA. **c** Comparison of BstXI restriction resistance levels in RcFAH12 PCR amplicons derived from leaf DNA of parental CL37 (lanes 1 and 2) or E720 T_2_ #11 (lanes 3 and 4). Lanes 1 and 3 represent uncut PCR products, samples in lanes 2 and 4 were digested with BstXI prior to gel electrophoresis. M = molecular weight marker

Control CL37 plants produced ~ 20% HFA, whereas eight of the nine *sgRNA*/*Cas9* combinations generated statistically significant reductions in average seed HFA content (unpaired Student’s *t* test, *p* values ranging from < 0.0001 to 0.0202) (Fig. 2). Two constructs were most effective: plasmid E719 (*UBQ10p:sgRNA* + *ACT8p:Cas9*) and plasmid E720 (*UBQ10p:sgRNA* + *YAOp:Cas9*), with most or all T_2_ seed samples containing less HFA than even the lowest CL37 control sample. E720 was particularly effective; the average HFA value for the set of 38 independent transgenic events was approximately half of the CL37 control average, with several lines reduced to < 5% HFA. Two lines contained only trace amounts of HFA (< 1%), representing near-homozygous *FAH12* gene knockout in a single generation. The reduction in HFA content could be directly linked to *FAH12* sequence alterations; representative copies of the gene showed typical types of insertions and deletions near the target site (Supplementary Fig. 4). Many of the mutations created in this line were also heritable, not solely the result of mutations generated anew in the T_2_ seeds. This was shown by planting multiple brown seeds from the segregating T_2_ seed pool to generate ‘non-transgenic’ lines lacking the *sgRNA*/*Cas9* T-DNA and its associated red fluorescence. All four lines examined lost HFA production in the resulting T_3_ seeds (Fig. 3b) while maintaining a homozygous mutant edited allele of the *RcFAH12* transgene (Fig. 3c). Six other E720 T_2_ lines containing strong seed HFA reductions (circled in Fig. 3a) showed similar outcomes (Supplementary Fig. 5). In all, five of the seven lines tested produced T_3_ seeds containing heritable gene editing events that resulted in homozygous *FAH12* mutant, *Cas9*-free status. Many of the other individuals in these data sets contained reduced levels of HFAs, relative to CL37 parental samples (< 17–20%), indicating lower levels of gene editing, but which could potentially result in a similar ‘finished line’ genetic status in T_4_ or later generations.

### Protospacer GC content alone is not predictive for gene editing efficiency

**Fig. 4:**
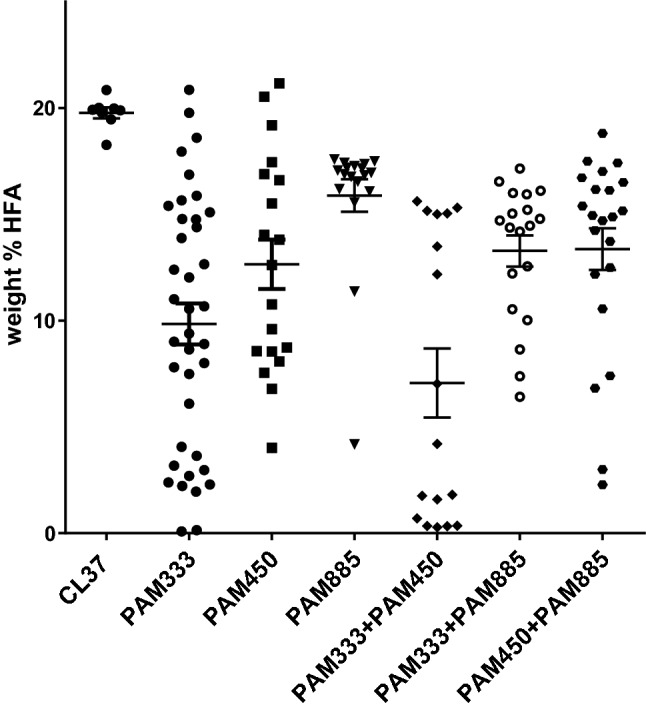
Testing the effect of protospacer GC content on RGR-type sgRNA efficacy. HFA levels in segregating T_2_ seed samples derived from *Arabidopsis* CL37 plants transformed with CRISPR constructs containing *YAO* promoter-driven Cas9 (Yan et al. 2015) and *AtUBQ10* promoter-driven sgRNAs targeting the region proximal to nucleotide positions 333, 540, or 885 of the *RcFAH12* ORF, or combinations thereof. The x-axis lists the targeted region, while the Y-axis represents the weight% of the HFAs present in each seed sample. Each data point represents the seeds from an individual transgenic T_1_ event. The bars in each data set represent the average and standard error of measurement. The unmodified CL37 control samples and the PAM333 samples are the same as those shown in Fig. 2 (CL37 and UBQ10p-sgRNA + YAOp-Cas9, respectively)

Protospacer GC nucleotide content often has also been addressed as an important aspect of sgRNA performance in various systems (Ren et al. 2014), including in plants (Morineau et al. 2017). We generated other CRISPR constructs that target different target sites in *RcFAH12* containing different GC content. The 20-bp protospacer preceding PAM333 targeted in Fig. 2 is 55% GC, while the two additional protospacers which target PAMs 540 and 885, contain 45% and 60% GC content, respectively. The specific sequences of the different *FAH12* protospacers and associated PAMs are shown in Supplementary Table 1. GC analysis of T_2_ seed samples produced from randomly chosen T_1_ plants revealed a range of HFA contents, which are compared in Fig. 4 to the parental CL37 and initial PAM333 data from Fig. 2. On average, the construct targeting PAM540 was nearly as effective as PAM333, with several lines containing < 10% HFA, although none < 3%. Conversely, the construct targeting PAM885 showed significantly less activity overall, with most individual samples containing only slight reductions in HFA content relative to the CL37 controls. We were also interested to study the effects of stacking together multiple sgRNAs targeting different sites within the same gene. The seed fatty acid profiles derived from lines combining the PAM885 sgRNA with PAMs 333 or 540 did not contain additional HFA reductions on average compared to the lines expressing single sgRNAs for PAM333 and PAM540, likely reflecting the relatively less effective activity conferred by the sgRNA targeting PAM885. However, more than half of the lines expressing the combination of sgRNAs for PAM333 and PAM540 had seed lipid profiles containing < 2% HFA, suggesting an additive gene editing effect when targeting multiple sites within the same gene (Fig. 4).

### Imperfect protospacer matches can still result in Cas9 activity

An sgRNA molecule carrying 20-bp protospacer that targets a DNA sequence, immediately adjacent to an 5′-NGG-3′ PAM sequence, should be exceptionally specific (theoretically occurring at random once every ~ 1.76 × 10^13^ bp). Yet, depending on the processes used to search for it, the degree of ‘off-site’ or ‘off-target’ Cas9 activity varies considerably from study to study (Feng et al. 2014; Tang et al. 2016; Zhang et al. 2018). Given the important implications of this effect, we sought to test for artificially induced off-target activity using the original *FAH12* PAM333 plasmid construct, by modifying it to contain a series of ten different single base-pair mismatches in the protospacer region, beginning at − 2 (two bp from the 3′ end of the 20 bp protospacer, proximal to the PAM sequence), and ending at position − 20 (distal to the PAM). A complete listing of the different protospacer sequences is shown in Table 1. As in previous experiments, CL37 plants were re-transformed with this series of constructs, followed by sowing of randomly chosen transgenic T_1_ seeds, harvesting of seeds from T_1_ plants, and analysis of T_2_ seed lipid HFA content by GC.

**Table 1:**
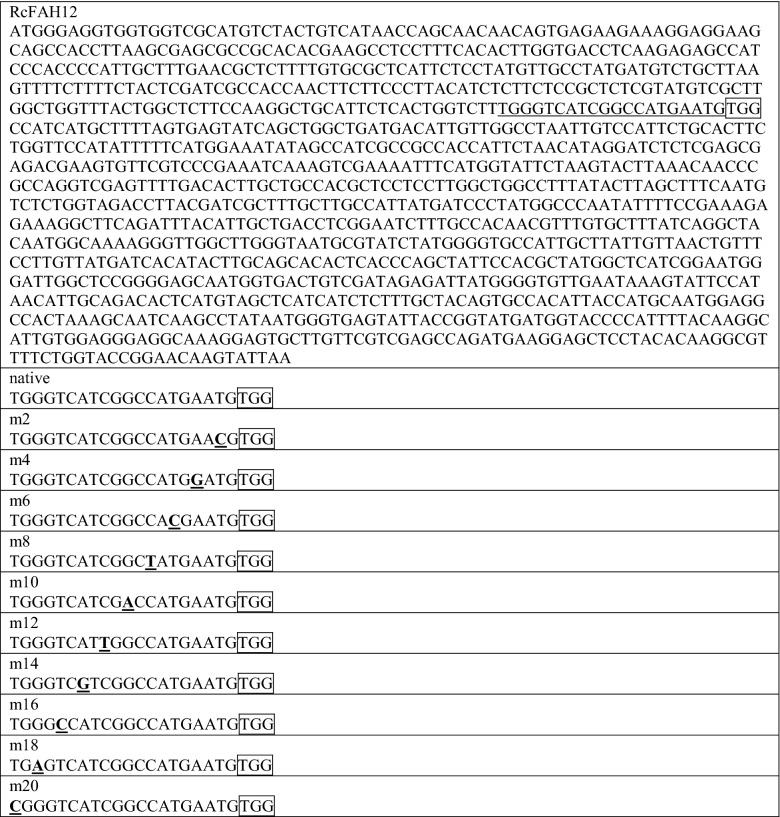
Nucleotide sequence of *RcFAH12* ORF expressed in *A. thaliana* CL37

PAM333 is boxed, and the protospacer is underlined. Lower rows show the native protospacer and PAM sequence, followed by the series of protospacers containing single bp mutations introduced in the sgRNA module (in bold and underlined), as discussed in Fig. 4

**Fig. 5:**
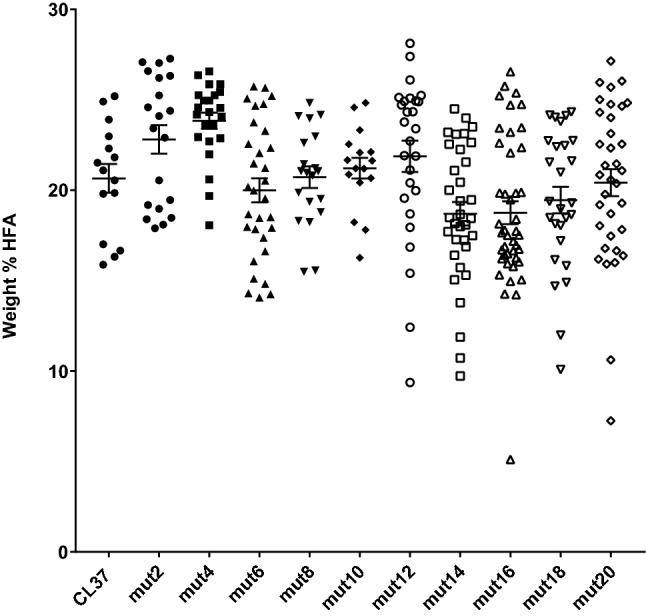
Assessment of the correlation between imperfectly matched sgRNA protospacer sequences and heritable changes to CL37 seed HFA levels. *Arabidopsis* CL37 plants were transformed with constructs containing *YAO* promoter-driven Cas9 paired with a series of *AtUBQ10* promoter-driven *RcFAH12* PAM333 sgRNAs, each containing a single mismatch to the target sequence (see also Table 2). The X-axis lists the location of each mutation, relative to the location of the first base of the PAM (e.g. m2 = mutation at position − 2, m4 = mutation at position − 4, etc.), while the Y-axis represents the weight% of the HFAs present in each seed sample. Each data point represents the seeds from an individual transgenic T_1_ event. The bars in each data set represent the average and standard error of measurement. Unmodified CL37 control samples are included

As shown in Fig. 5, the control CL37 plants grown for this experiment ranged between 18 and 26% with an average of ~ 21% HFA. The m4 construct produced a dataset that was significantly different than the CL37 controls (Student *t* test, *p* = 0.0006), but in this case, the average HFA value was *higher* than the controls. All other mutant protospacer constructs produced lines with seed HFA levels that were statistically indistinguishable from the controls, on average. However, each mutant series produced at least a few individual lines with seed HFA levels below the range of values in the CL37 controls (Fig. 5).

**Fig. 6:**
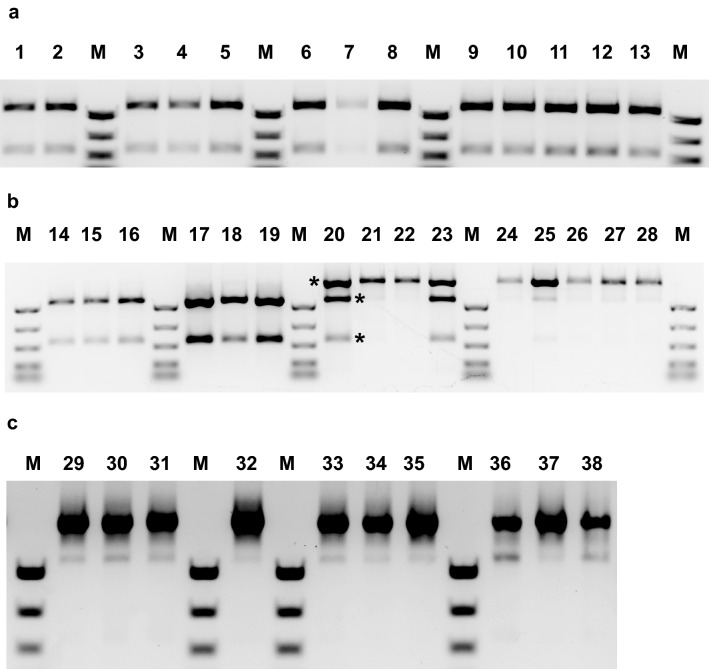
Assessment of the correlation between changes in DNA-level gene editing and seed HFA content. T_2_ seedlings grown from selected lines representing the lowest values from each of the mutant series shown in Fig. 4 were used to isolate genomic DNA, which was then pre-treated with BstXI to reduce unedited transgenic *RcFAH12* copy number. The *RcFAH12* ORF was then amplified by PCR, treated again with BstXI, followed by fragment separation by agarose gel electrophoresis to compare relative levels of edited and unedited DNA. The locations of the 1164 bp full-length PCR product and the 832 and 332 bp BstXI digestion products not shown) are marked by descending asterisks in lane 20. M = molecular weight marker (PCR marker, New England Biolabs). **a** Lanes 1–2—CL37 parental controls, 20.6% and 21.6% HFA, respectively. Lane 3–5—m2 series, lines #14, 17, and 20: 17.9%, 18.1%, and 18.5%, respectively. Lanes 6–8—m4 series, lines #1, 2, and 3: 22.0%, 20.6%, and 18.1%, respectively. Lanes 9–13—m6 series, lines #7, 8, 13, 16, and 23: 14.2%, 16.0%, 14.8%, 14.3%, and 14.1%, respectively. **b** Lanes 14–16—m8 series, lines #2, 5, and 17: 18.2%, 15.6%, 15.5% HFA, respectively. Lanes 17–19—m10 series, lines #3, 4, and 12: 17.8%, 16.3%, and 18.2% HFA respectively. Lanes 20–23—m12 series, lines #10, 12, 17, and 27: 15.4%, 12.4%, 9.4%, and 16.9% HFA, respectively. Lanes 24–28—m14 series, lines #6, 13, 16, 25, and 26: 9.7%, 11.9%, 10.7%, 13.8% and 15.7% HFA, respectively. **c** Lanes 29–31—m20 series, lines #12, 14, and 29: 16.2%, 10.6%, 7.2% HFA, respectively. Lane 32—m16 series, line #10, 5.1% HFA. Lanes 33–35—m18 series, lines #11, 14, 21: 14.9%, 12.0%, and 14.7% HFA, respectively. Lanes 36–38—< 1% HFA native PAM333 control lines (Fig. 3)

We tested the genomic DNA present in these low-HFA seed samples for CRISPR modifications at the target site in the *FAH12* gene. Samples of genomic DNA were isolated from pooled T_2_ seedlings derived from at least one reduced-HFA line from each of the m2 through m20 sgRNA protospacer mutant series shown in Fig. 5. Samples were digested with BstXI, then 10 ng of restricted DNA was used as template for *FAH12* PCR. These amplicons were again digested with BstXI, then analyzed by agarose gel electrophoresis. BstXI was used here due to proximity of a restriction site near PAM333 (Supplementary Fig. 4). Unmodified plants were included as negative controls, while samples from < 1% HFA E720 lines (shown in Fig. 2) were included as editing-positive controls (Fig. 6). Some BstXI sites in the genomic samples were not cleaved during the pre-treatment step, as indicated that unedited, BstXI-sensitive *FAH12* templates from control plants and the m2 through m10 protospacer mutant lines containing the lowest HFA values survived the enzymatic pre-treatment, thus generating *FAH12* amplicons that were completely or substantially digested by the second BstXI treatment (Fig. 6a, all lanes; Fig. 6b, lanes 14–19). These data suggest that few if any mutations had occurred in or had been transmitted to the seed tissues of the m2, m4, m6, m8, or m10 lines contained near-normal HFA levels and that the modest variances in HFA content in these samples, relative to CL37 controls, arose through typical biological variability.

However, seeds from plants expressing sgRNAs containing protospacers with increasingly PAM-distal mismatches (e.g. m12 through m20) showed a general downward trend in average HFA levels and individual samples with progressively larger decreases. The apparent tolerance for mismatches between sgRNA and target DNA increases beginning at positions − 12/− 14, and the relative drop in HFA production correlated well with the levels of *BstX*I-resistant *FAH12* DNA that survived the enzymatic pre-treatment (see increased ratio of *BstX*I resistant/BstXI-sensitive PCR digestion, Fig. 6b, lanes 20–28, and all lanes in Fig. 6c). These data indicate that perfect pairing of sgRNA to target site DNA is not an absolute requirement to achieve occasional gene editing events in plant cells, thus emphasizing the need to design sgRNAs carefully, and to remain aware of potential ‘off-target’ activity.

**Fig. 7:**
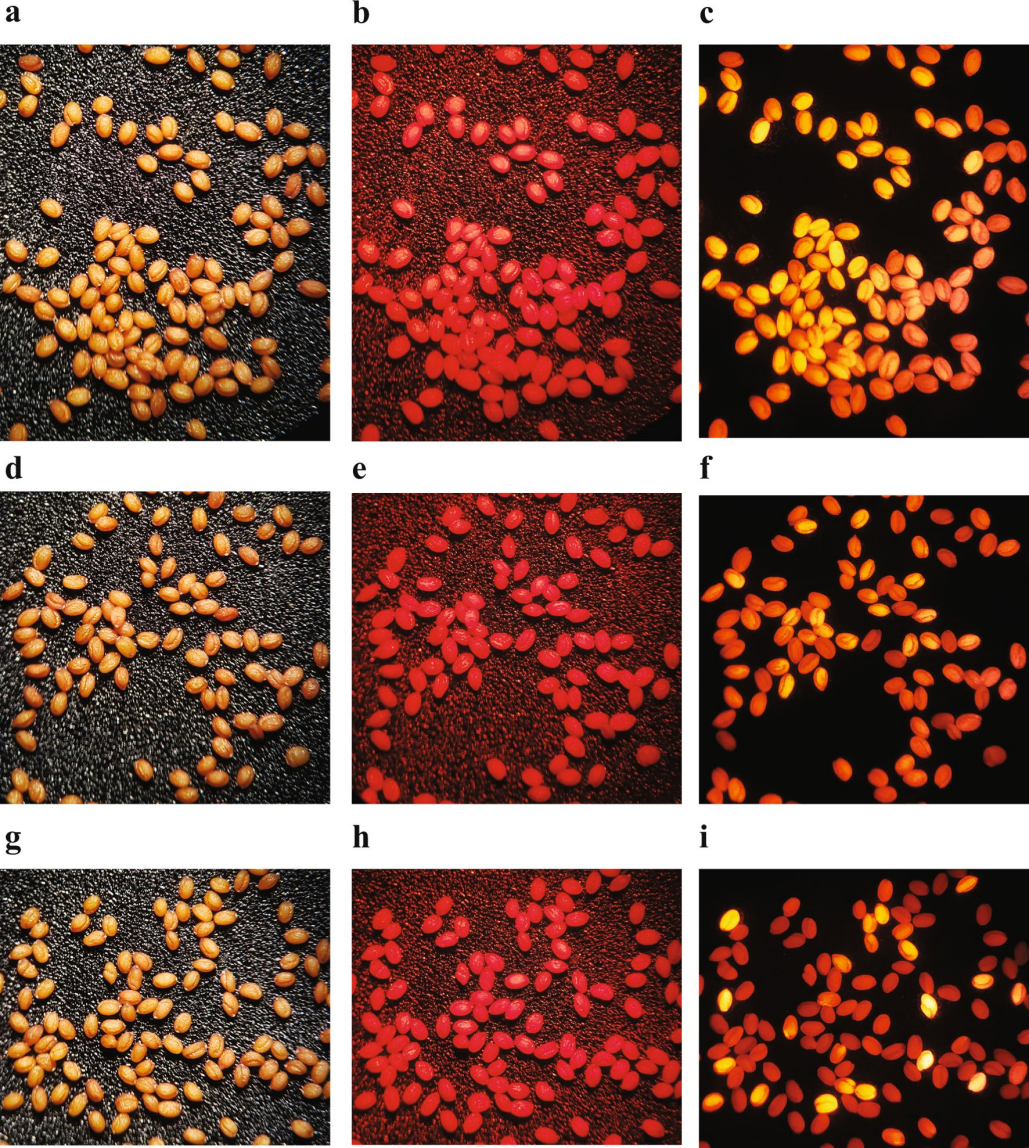
Visual inspection of fluorescence levels in T_2_ seeds of *Arabidopsis* E113 plants expressing efficacious *DsRed*-targeted gene editing constructs. The active *sgRNA* from construct E642 used to target the *DsRed* gene in vegetative tissues described in Fig. 1 was fused to the *UBQ10* promoter and combined with the *YAOp:Cas9* cassette. The resulting construct (called E767) was transformed into *Arabidopsis* E113 and basta herbicide-resistant seedlings were selected and grown to maturity. Segregating T_2_ seed samples from three representative lines were inspected visually by illumination with normal light (**a**, **d**, **g**), green light with no red filter (**b**, **e**, **h**) and green light with red filtering (**c**, **f**, **i**)

Finally, we tested the efficacy of the *UBQ10p*:*sgRNA* + *YAOp:Cas9* plasmid binary architecture to re-address the failed ability to create transmissible gene editing events in the *DsRed* transgene in *Arabidopsis* E113 seeds, as discussed above (Fig. 1 and Supplementary Figs. 2 and 3). The *DsRed sgRNA* targeted to position 422 (used in construct E642, Fig. 1) was transferred to the *UBQ10p*-based sgRNA module and combined into the *YAOp:Cas9* binary plasmid construct, and transformed into *Arabidopsis* E113. T_1_ seedlings were selected for glufosinate resistance, and grown to maturity. T_2_ seeds were harvested from multiple lines for each construct and inspected visually. Control construct E716, lacking a *sgRNA* element, continued to produce uniform red fluorescence patterns (Supplementary Fig. 6). However, unlike the plasmid designs containing *nos*/*CaMV35S* promoters, the *UBQ10p:DsRedsgRNA*/*YAOp:Cas9* combination in construct E767 demonstrated high levels of activity in reproductive tissues. T_2_ seed samples harvested from 28 independent transgenic events revealed 8 lines that contained seeds with visually obvious levels of red fluorescence depletion. The levels of seeds edited at scale varied from 1.4 to 69.8%, with an average of 28%. Figure 7 shows examples of the fluorescence patterns of samples containing low (Fig. 7c), moderate (Fig. 7f), and high (Fig. 7i) levels of *DsRed* gene editing.

## Discussion

CRISPR technology has provided avenues for engineering of cells, tissues, and whole organisms across the biological spectrum. Aside from the amazing potential for gene editing as a diagnostic tool and perhaps even a treatment for many debilitating diseases (Ortiz-Virumbrales et al. 2017; Reczek et al. 2017; Zabinyakov et al. 2017), the potential possible uses of this technology in plants and livestock animals is similarly impressive (Lamas-Toranzo et al. 2017). Creation of disease-resistant and less allergenic food crops (Hummel et al. 2018; García-Molina et al. 2019) with enhanced nutrient profiles (Wang et al. 2019) is now within our reach; the future implications for feeding a progressively larger world population are immense.

Many research groups around the world have developed extensive gene editing toolkits. We are not seeking to ‘compete’ in this regard, in fact, we will likely utilize some of the resources developed by these research groups (Lowder et al. 2015; Čermák et al. 2017; Castel et al. 2019) in the future. We simply have tried to address some of the important criteria, such as regulatory element choice, and consideration of how sgRNA design affects both ‘on-target’ and ‘off-target’ gene editing, that must be considered before adopting this new technology. These topics are often not described in detail in studies of individual edited genes, or are presented as small additions to complex analyses of large data sets, or are referred to abstractly as data points used to build predictive computer algorithms. Here we established facile phenotypic screens that were used to evaluate different variables in the CRISPR design process using simple, easily screened test cases. It is our hope that other research teams may be able to apply these principles to their genes of interest, and CRISPR strategies to edit them, as well.

Many existing CRISPR studies in plants have made use of the RNA polymerase III class of promoters for expression of the sgRNA component. While often very successful, this type of promoter has some limitations, including specific sequence contexts that must be maintained at the 5′ end of the transcript (Gao et al. 2017), and the length of transcripts that can be produced. Our future plans for applying CRISPR technology to oilseed engineering dictates that we will likely need to simultaneously edit multiple genes. Lowder et al. (2015) established a system that allows for packaging multiple RNApolIII promoter:sgRNA units together in series, but the sgRNA transcript sequence limitations (Gao et al. 2017) still remain. Gao and Zhao (2014) utilized the ability of certain ribozyme RNA sequences to self-splice to create new sgRNA production modules. This approach, along with other RNA processing-related approaches (Xie et al. 2015; Čermák et al. 2017), makes possible the expression of several different sgRNAs from one plasmid, using any RNA polymerase type II promoter. Our constructs rely on production of mature sgRNAs from expression modules containing the specific sgRNA sequence flanked on the 5′ and 3′ sides by hammerhead and HDV ribozymes (Table 2).

**Table 2:**
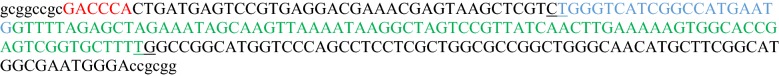
Structure and basic sequence components of ‘RGR’ self-splicing ribozyme guide RNA gene sequence

The 6 bp sequence that participates in the formation of the H1 hairpin required for hammerhead self-splicing is shown in red. This sequence is the reverse complement of the first 6 bp of the protospacer sequence (Gao and Zhao 2014). The remainder of the hammerhead ribozyme sequence is shown in black, 3′ to the H1 hairpin sequence. The 20 bp protospacer sequence is shown in blue, and is linked to the remainder of the mature guide RNA sequence shown in green font. The HDV ribozyme sequence is shown in black font, 3′ to the mature sgRNA. The RNA self-splicing target sites are underlined. NotI and SacII restriction sites (shown in lower case font) at the 5′ and 3′ ends, respectively, are used for cloning

Expression of *nos* promoter-driven plant codon-optimized SpCas9 with either of two different sgRNAs expressed behind the *CaMV35s* promoter (Bevan 1984; Gleave 1992) both effectively targeted the *DsRed* selectable marker gene in a transgenic *Arabidopsis* line (Fig. 1). This result was encouraging as a proof of concept, and seemed to suggest that the ribozyme-based sgRNA design works effectively, in contrast to the relatively poor performance of ribozyme sgRNA reported recently (Čermák et al. 2017). The use of strong plant pathogen-derived promoters such as *CaMV35s* might be ideal for single-generation studies of gene editing effects in vegetative tissues. But as in previous studies (Shan et al. 2018) the strong editing activity from these designs did not result in meaningful numbers of heritable mutations, thus precluding the continued use of this set of regulatory elements in our future studies.

To fully exploit the utility of gene editing as an oilseed metabolic engineering tool, we conducted direct comparisons of promising promoters that could act in reproductive cells and tissue types, to generate heritable mutations. A few candidate promoters had been previously described and characterized in some detail (Wang et al. 2015; Yan et al. 2015; Zhang et al. 2016). We cloned these and other promoters and used them to assess their relative abilities to create heritable mutations leading to reduced HFA production in T_2_ generation *Arabidopsis* CL37 seeds (Lu et al. 2006) via editing of the *RcFAH12* fatty acid hydroxylase gene. In addition, we also tested the *Arabidopsis actin8* (*AtACT8*) promoter (An et al. 1996), which we previously confirmed is expressed at high levels in *Arabidopsis* flowers and developing seeds (Shockey et al. 2003). As shown in Fig. 2, most of the different combinations of promoters fused to Cas9 and sgRNA did effect slight, but statistically meaningful, reductions in T_2_ seed HFA levels. In our system, the enhanced egg cell-specific promoter (*EC1.2en-EC1.1p*) did not produce highly significant HFA reductions in T_2_ seeds, unlike the reported ability of this promoter to drive high mutation rates in other genes in the first generation (Wang et al. 2015). The cause of this discrepancy is not known. This promoter (and *rbcS* terminator) used here is the same as that contained in construct pHEE2E-TRI, which demonstrated high activity against other *Arabidopsis* genes (Wang et al. 2015). One or two copies of this promoter were included in five of the nine different binary constructs tested in Fig. 2; four of these five did show slight but statistically significant decreases in seed HFA content relative to CL37 controls. It is very possible that some of the individual lines chosen from these populations could produce higher levels of heritable gene editing events in T_3_ or later generation seed samples.

Čermák et al. (2017) have presented compelling evidence that ribozyme-based sgRNA processing is not as effective as some other sgRNA production methods, such as those that rely on the transfer RNA processing enzymes. We have not directly compared the tRNA system to the ribozyme system employed here, but our data (Fig. 2) seemed fairly robust, so we continued to employ the ribozyme design.

Only two combinations led to individual samples containing < 15 weight% HFA levels, which would represent loss-of-function mutations in at least ~ 25% of oil-producing T_2_ seed cells. Both highly active constructs contained *sgRNA* expressed behind the *Arabidopsis ubiquitin 10* (*AtUBQ10*) promoter (Zhang et al. 2016), combined with *Cas9* expression driven by either the *AtACT8* promoter (construct E719) (An et al. 1996) or the *YAO* promoter (construct E720) (Yan et al. 2015). Construct E720 was highly active, producing many lines that contained strong reductions in HFA content, including two that approached near-saturation of *FAH12* editing (as evidenced by only trace amounts of HFA remaining in segregating T_2_ seeds). These data also confirm the findings from Fig. 1 that the ribozyme-based sgRNA production process is reasonably efficient and rapid, but improves upon the results shown in Fig. 1 in that many of the mutations produced are heritable, and can results in significant numbers of homozygous edited non-transgenic lines as early as the T_2_ and T_3_ generations (Fig. 3, Supplementary Fig. 5). The results also show that the *AtACT8* promoter, which had not been analyzed in previously reported plant CRISPR studies, can be an effective element in future gene editing strategies as well.

The complex set of factors that control sgRNA targeting efficiency remain a vexing problem. Previous studies have identified certain sequence elements within the protospacer that may influence Cas9 biochemical outcomes. Many reveal the importance of protospacer GC content generally, as a component of sgRNA melting temperature, but also in specific regions of the protospacer. These include guanine at position − 1 relative to the PAM (Wong et al. 2015) and overall GC content in PAM-proximal and PAM-distal sections of the protospacer (Labuhn et al. 2018). Based on guidance derived by Ren et al. (2014), Morineau et al. (2017) achieved successful gene-editing in *Camelina sativa* with protospacer sequences that include guanine or cytosine in at least 5 of the 6 bp proximal to the PAM. Yet, in Fig. 4, we show that sgRNAs targeted to *RcFAH12* PAM885, which contains the highest overall protospacer GC content (and 5 of 6 guanines or cytosines from positions − 6 to − 1, including G at position − 1), was much less efficient than PAM 333 (which contains − 1G but only 2 of 6 guanine-cytosines from − 6 to − 1) or PAM540, which contained the lowest overall protospacer GC content and contained a cytosine at position − 1, not a guanine. These results suggest that much work remains to be done to develop reliable sgRNA design prediction algorithms, and that most research groups should still plan to test multiple potential target sites in their genes of interest when initiating new CRISPR studies.

The other serious concern regarding CRISPR technology is the risk of ‘off-target’ activity in genes not specified by the designated sgRNAs. Our results confirm findings presented in many other past studies that the ‘seed region’ (the 10–12 bp proximal to the PAM) confers the majority of specificity to sgRNA binding and Cas9 recruitment (Wilson et al. 2018), given the absence of meaningful mutation rates in CL37 lines transformed with sgRNAs containing target sites mismatches in the PAM-proximal half of the protospacer (Fig. 5, m2 through m10). However, at-scale mutations did begin to appear with increasing frequency when analyzing the effects of sgRNA constructs containing mismatches at increasing distances from the PAM (Fig. 5, m12 through m20). How much of a problem is off-site editing? Extant results, both here and in previous studies, suggest that the specificity of CRISPR-Cas9 based gene editing can be somewhat flexible, at least in some cases. In this sense, the power of gene editing technology may create risks, but the ability to manipulate this flexibility may also present opportunities. Generally speaking, Cas9 nuclease activity is only directed to intended target sites specified by the protospacer region of the sgRNA. Given the massive proliferation of sequenced genomes throughout all branches of the tree of life in recent years, it is increasingly easy to perform checks of any candidate protospacer sequence against all possible intended and closely related unintended targets. Yet, in some cases, researchers may wish to target more than one closely related gene for editing. Nearly 17% of all *Arabidopsis thaliana* genes exist as related orthologs in closely linked tandem arrays (The Arabidopsis Genome Initiative 2000) and many genes in practically all other sequenced plant genomes are also part of large gene families distributed across syntenic chromosomal regions that arose during evolution (Shockey and Browse 2011). Creation of lines containing mutations in multiple genes via the use of intentionally promiscuous sgRNA constructs may be an attractive approach for some researchers, especially in cases where functional overlap between related genes masks the effects of mutations in a single member of a gene family.

In summary, we have presented here the results of a series of experiments designed to reveal which promoter elements and sgRNA design criteria may help to achieve stable, heritable mutations in *Arabidopsis* and related plant species, accompanied by analyses that provide an estimate of how often off-target mutations might occur. These tools are freely available to the public and can be easily combined with other existing resources (Shockey et al. 2015) to combine gene editing with transgene overexpression and/or partial silencing of endogenous genes.

## Methods

### Gene cloning and plasmid construction

The makeup of the basic set of cloning vector and plant transformation binary plasmids has been described previously (Shockey et al. 2015). A plasmid bearing the open reading frame for epitope-tagged, plant codon-optimized *Staphylococcus aureus* Cas9 was generously provided by Dr. Jen Sheen (Harvard Medical School) (Li et al. 2013). The DNA templates for sgRNA production utilized the self-cleaving ribozyme ‘Ribozyme-Guide RNA-Ribozyme’ (RGR) design (Gao and Zhao 2014). The sequence for the representative *RcFAH12* PAM333 construct is described in Table 1. Synthetic versions of the promoters used in this study were purchased commercially (see Supplementary Table 2 for sequences), based on the sequences reported by the specific authors who first described them. These were cloned into existing cloning vectors (Shockey et al. 2015) to replace the existing promoters. Single-guide RNA genes were either purchased commercially, or adapted to modify existing target sequences by mutagenesis PCR. Multiple RGR sgRNAs were combined in series by generating restriction-digested sgRNA units that had been PCR-amplified with nucleotide primers containing unique BstXI restriction sites at the appropriate internal junctions, and NotI or SacII sites at the extreme 5′ and 3′ ends, respectively. Purified digested single or pooled sgRNA cassettes were ligated into the cloning vectors K34 or K63, and the resulting promoter:sgRNA:terminator cassettes were combined with similarly prepared Cas9 expression cassettes for ligation into plant binary plasmids B9 or B110 using simple restriction digestion and T4 DNA ligase-based molecular techniques (Shockey et al. 2015). All paired *sgRNA* and *Cas9* expression modules were constructed in the head-to-head divergent orientation to maximize transcription of both elements (Castel et al. 2019). Complete plasmid maps of all constructs described in this study are available upon request.

### Plant growth and transformation

Most of the studies described here utilized *Arabidopsis thaliana* CL37 (Lu et al. 2006). CL37 is derived from the *fae1* mutant, which is defective in 18-carbon fatty acid elongation (Kunst et al. 1992), and overexpresses the *Ricinus communis FAH12* oleate hydroxylase gene (van de Loo et al. 1995) in its seeds. All binary plasmids were prepared using standard molecular biology cloning techniques and electroporated into transformation-competent *Agrobacterium tumefaciens* strain C58-C1, then transformed into *Arabidopsis* using the floral dip procedure (Clough and Bent 1998).

### Seed lipid analysis

Fatty acid methyl esters (FAMEs) were produced from intact seeds, by incubating ~ 20–50 mg of seeds in 1.5 ml of 5% sulfuric acid in methanol at 85–90 ℃ for 60–90 min. The reactions were quenched with 1.5 ml of saturated NaCl solution, and mixed with 400–800 μl of hexane, followed by vigorous mixing and centrifugation at 2500×*g* for 5 min. Hexane fractions from clarified samples were analyzed for resolution of all FAMEs, including those from hydroxy fatty acids (HFAs), by GC as previously described (Shockey et al. 2019).

## Electronic supplementary material

Below is the link to the electronic supplementary material.

Supplementary material 1 Supplementary Fig. 1 Examples of specific types of Cas9-mediated mutagenesis in vegetative tissues of *Arabidopsis* E113. Genomic DNA was isolated from fluorescence-depleted T_1_ leaf tissue of E640 and E642 plants, the *DsRed* ORF amplified by PCR, and digested with *Nco*I or *Pst*I as appropriate. Restriction-resistant amplicons were purified, cloned, and sequenced. Representative examples are shown below, aligned to the native *DsRed* sequence. Query = native *DsRed*. Subject = cloned sequence from E640/E642 CRISPR plants (DOCX 24 kb)

Supplementary material 2 Supplementary Fig. 2 Visual inspection of fluorescence levels in T_2_ seeds of *Arabidopsis* E113 plants expressing negative control gene editing constructs lacking *sgRNA* elements. Segregating T_2_ seeds produced from 64 independent T_1_ lines expressing construct E638 used as a control to monitor parental red fluorescence in vegetative tissues (see Fig. 1) were harvested and inspected for changes in seed fluorescence levels. Samples from three representative lines were inspected visually by illumination with normal light (a, d, g), green light with no red filter (b, e, h) and green light with red filtering (c, f, i) (EPS 1,58,732 kb)

Supplementary material 3 Supplementary Fig. 3 Visual inspection of fluorescence levels in T_2_ seeds of *Arabidopsis* E113 plants expressing *DsRed*-targeted gene editing elements driven by constitutive promoters. Segregating T_2_ seeds produced from 64 independent T_1_ lines expressing either construct E640 or E642 used to target the *DsRed* gene in vegetative tissues (see Fig. 1 and Supplementary Fig. 1) were harvested and inspected for changes in parental red fluorescence. Samples from three representative E642 lines were inspected visually by illumination with normal light (a, d, g), green light with no red filter (b, e, h) and green light with red filtering (c, f, i). (EPS 1,55,463 kb)

Supplementary material 4 Supplementary Fig. 4 Mutant *FAH12* loci CRISPR CL37 plants display typical types of Cas9-generated mutations. Cas9-mutated *FAH12* PCR amplicons were produced by PCR, from genomic DNA isolated from E719 (*UBQ10p:sgRNAAtACT8p:Cas9*) and E720 (*UBQ10p:sgRNA* + *YAOp:Cas9*) plants that contained reduced amounts of seed HFA. After treatment with *BstX*I, restriction-resistant PCR products were purified, cloned, and sequenced. Seventeen copies were analyzed, which included: 9 single bp insertions, 3 single-bp deletions, two 4-bp deletions, one 6-bp deletion, one 7-bp deletion, and one 12-bp deletion. Representative sequences are shown below (subject), aligned to native *FAH12* sequence (query). The PAM333 sequence is underlined, the recognition sequence for *BstX*I used to screen for mutations is boxed in yellow. See Fig. 2 for more details (DOCX 15 kb)

Supplementary material 5 Supplementary Fig. 5 Assessment of heritability of mutations acquired in additional low-HFA CL37 lines transformed with hydroxylase gene editing constructs. See Fig. 3 for analysis of segregating T_2_ seeds from line E720 #11, which contains <0.1% HFA. Other low-HFA samples circled in Fig. 3A were used to plant brown T_2_ seeds lacking gene editing T-DNA; HFA levels in T_3_ seeds from multiple progeny lines were quantified by GC. The HFA content in each segregating T_2_ seed sample is shown in parentheses following the line number in the title of each panel. (EPS 314 kb)

Supplementary material 6 Supplementary Fig. 6 Visual inspection of fluorescence levels in T_2_ seeds of *Arabidopsis* E113 plants expressing negative control gene editing constructs. Binary construct E716 (containing only a *YAOp:Cas9* cassette) was transformed into *Arabidopsis* E113 and glufosinate-resistant seedlings were selected and grown to maturity. This construct lacks the *DsRed*-specific sgRNA element used in the lines shown in Fig. 7. Segregating T_2_ seed samples from three representative lines were inspected visually by illumination with normal light (a, d, g), green light with no red filter (b, e, h) and green light with red filtering (c, f, i). (EPS 1,74,898 kb)

Supplementary material 7 (DOCX 13 kb)

Supplementary material 8 (DOCX 14 kb)

## References

[CR1] An YQ, McDowell JM, Huang S, McKinney EC, Chambliss S, Meagher RB (1996). Strong, constitutive expression of the *Arabidopsis* ACT2/ACT8 actin subclass in vegetative tissues. Plant J.

[CR2] Bevan M (1984). Binary *Agrobacterium* vectors for plant transformation. Nucleic Acids Res.

[CR3] Butler NM, Baltes NJ, Voytas DF, Douches DS (2016). Geminivirus-mediated genome editing in potato (*Solanum tuberosum* L.) using sequence-specific nucleases. Front Plant Sci.

[CR4] Castel B, Tomlinson L, Locci F, Yang Y, Jones JDG (2019). Optimization of T-DNA architecture for Cas9-mediated mutagenesis in *Arabidopsis*. PLoS ONE.

[CR5] Čermák T, Curtin SJ, Gil-Humanes J, Čegan R, Kono TJY, Konečná E, Belanto JJ, Starker CG, Mathre JW, Greenstein RL, Voytas DF (2017). A multipurpose toolkit to enable advanced genome engineering in plants. Plant Cell.

[CR6] Clough SJ, Bent AF (1998). Floral dip: a simplified method for *Agrobacterium*-mediated transformation of *Arabidopsis thaliana*. Plant J.

[CR7] Dyer JM, Chapital DC, Kuan J-C, Mullen RT, Turner C, McKeon TA, Pepperman AB (2002). Molecular analysis of a bifunctional fatty acid conjugase/desaturase from tung. Implications for the evolution of plant fatty acid diversity. Plant Physiol.

[CR8] Feng Z, Mao Y, Xu N, Zhang B, Wei P, Yang DL, Wang Z, Zhang Z, Zheng R, Yang L, Zeng L, Liu X, Zhu JK (2014). Multigeneration analysis reveals the inheritance, specificity, and patterns of CRISPR/Cas-induced gene modifications in *Arabidopsis*. Proc Natl Acad Sci USA.

[CR9] Gao Y, Zhao Y (2014). Self-processing of ribozyme-flanked RNAs into guide RNAs in vitro and in vivo for CRISPR-mediated genome editing. J Integr Plant Biol.

[CR10] Gao Z, Harwig A, Berkhout B, Herrera-Carrillo E (2017). Mutation of nucleotides around the +1 position of type 3 polymerase III promoters: the effect on transcriptional activity and start site usage. Transcription.

[CR11] García-Molina MD, Giménez MJ, Sánchez-León S, Barro F (2019). Gluten free wheat: are we there?. Nutrients.

[CR12] Gilbert LA, Larson MH, Morsut L, Liu Z, Brar GA, Torres SE, Stern-Ginossar N, Brandman O, Whitehead EH, Doudna JA, Lim WA, Weissman JS, Qi LS (2013). CRISPR-mediated modular RNA-guided regulation of transcription in eukaryotes. Cell.

[CR13] Gleave AP (1992). A versatile binary vector system with a T-DNA organisational structure conducive to efficient integration of cloned DNA into the plant genome. Plant Mol Biol.

[CR14] Haughn G, Somerville CR (1986). Sulfonylurea-resistant mutants of *Arabidopsis*. Mol Gen Genet.

[CR15] Hummel AW, Chauhan RD, Čermák T, Mutka AM, Vijayaraghavan A, Boyher A, Starker CG, Bart R, Voytas DF, Taylor NJ (2018). Allele exchange at the EPSPS locus confers glyphosate tolerance in cassava. Plant Biotechnol J.

[CR16] Jinek M, Chylinski K, Fonfara I, Hauer M, Doudna JA, Charpentier E (2012). A programmable dual-RNA-guided DNA endonuclease in adaptive bacterial immunity. Science.

[CR17] Kumlehn J, Pietralla J, Hensel G, Pacher M, Puchta H (2018). The CRISPR/Cas revolution continues: from efficient gene editing for crop breeding to plant synthetic biology. J Integr Plant Biol.

[CR18] Kunst L, Taylor DC, Underhill EW (1992). Fatty acid elongation in developing seed of *Arabidopsis thaliana*. Plant Physiol Biochem.

[CR19] Labuhn M, Adams FF, Ng M, Knoess S, Schambach A, Charpentier EM, Schwarzer A, Mateo JL, Klusmann JH, Heckl D (2018). Refined sgRNA efficacy prediction improves large- and small-scale CRISPR-Cas9 applications. Nucleic Acids Res.

[CR20] Labun K, Montague TG, Gagnon JA, Thyme SB, Valen E (2016). CHOPCHOP v2: a web tool for the next generation of CRISPR genome engineering. Nucleic Acids Res.

[CR21] Labun K, Montague TG, Krause M, Torres Cleuren YN, Tjeldnes H, Valen E (2019). CHOPCHOP v3: expanding the CRISPR web toolbox beyond genome editing. Nucleic Acids Res.

[CR22] Lamas-Toranzo I, Guerrero-Sánchez J, Miralles-Bover H, Alegre-Cid G, Pericuesta E, Bermejo-Álvarez P (2017). CRISPR is knocking on barn door. Reprod Domest Anim.

[CR23] Lei Y, Lu L, Liu HY, Li S, Xing F, Chen LL (2014). CRISPR-P: a web tool for synthetic single-guide RNA design of CRISPR-system in plants. Mol Plant.

[CR24] Li JF, Norville JE, Aach J, McCormack M, Zhang D, Bush J, Church GM, Sheen J (2013). Multiplex and homologous recombination-mediated genome editing in *Arabidopsis* and *Nicotiana benthamiana* using guide RNA and Cas9. Nat Biotechnol.

[CR25] Liang G, Zhang H, Lou D, Yu D (2016). Selection of highly efficient sgRNAs for CRISPR/Cas9-based plant genome editing. Sci Rep.

[CR26] Lowder LG, Zhang D, Baltes NJ, Paul JW, Tang X, Zheng X, Voytas DF, Hsieh T, Zhang Y, Qi Y (2015). A CRISPR/Cas9 toolbox for multiplexed plant genome editing and transcriptional regulation. Plant Physiol.

[CR27] Lu CF, Fulda M, Wallis JG, Browse J (2006). A high-throughput screen for genes from castor that boost hydroxy fatty acid accumulation in seed oils of transgenic *Arabidopsis*. Plant J.

[CR28] Marx V (2018). Base editing a CRISPR way. Nat Methods.

[CR29] McCourt P, Benning C (2010). *Arabidopsis*: a rich harvest 10 years after completion of the genome sequence. Plant J.

[CR30] Morineau C, Bellec Y, Tellier F, Gissot L, Kelemen Z, Noguè F, Faure JD (2017). Selective gene dosage by CRISPR-Cas9 genome editing in hexaploid *Camelina sativa*. Plant Biotechnol J.

[CR31] O’Malley RC, Ecker JR (2010). Linking genotype to phenotype using the *Arabidopsis* unimutant collection. Plant J.

[CR32] Ortiz-Virumbrales M, Moreno CL, Kruglikov I, Marazuela P, Sproul A, Jacob S, Zimmer M, Paull D, Zhang B, Schadt EE, Ehrlich ME, Tanzi RE, Arancio O, Noggle S, Gandy S (2017). CRISPR/Cas9-Correctable mutation-related molecular and physiological phenotypes in iPSC-derived Alzheimer's PSEN2 (N141I) neurons. Acta Neuropathol Commun.

[CR33] Piatek A, Ali Z, Baazim H, Li L, Abulfaraj A, Al-Shareef S, Aouida M, Mahfouz MM (2015). RNA-guided transcriptional regulation in planta via synthetic dCas9-based transcription factors. Plant Biotechnol J.

[CR34] Reczek CR, Birsoy K, Kong H, Martínez-Reyes I, Wang T, Gao P, Sabatini DM, Chandel NS (2017). A CRISPR screen identifies a pathway required for paraquat-induced cell death. Nat Chem Biol.

[CR35] Ren X, Yang Z, Xu J, Sun J, Mao D, Hu Y, Yang S-J, Qiao HH, Wang X, Hu Q, Deng P, Liu LP, Ji JY, Li JB, Ni JQ (2014). Enhanced specificity and efficiency of the CRISPR/Cas9 system with optimized sgRNA parameters in *Drosophila*. Cell Rep.

[CR36] Shan Q, Baltes NJ, Atkins P, Kirkland ER, Zhang Y, Baller JA, Lowder LG, Malzahn AA, Haugner JC, Seelig B, Voytas DF, Qi Y (2018). ZFN, TALEN and CRISPR-Cas9 mediated homology directed gene insertion in *Arabidopsis*: a disconnect between somatic and germinal cells. J Genet Genom.

[CR37] Shockey J, Browse J (2011). Genome-level and biochemical diversity of the acyl-activating enzyme superfamily in plants. Plant J.

[CR38] Shockey JM, Fulda MS, Browse J (2003). Arabidopsis contains a large superfamily of acyl-activating enzymes. Phylogenetic and biochemical analysis reveals a new class of acyl-coenzyme A synthetases. Plant Physiol.

[CR39] Shockey J, Mason C, Gilbert M, Cao H, Li X, Cahoon E, Dyer J (2015). Development and analysis of a highly flexible multi-gene expression system for metabolic engineering in *Arabidopsis* seeds and other plant tissues. Plant Mol Biol.

[CR40] Shockey J, Lager I, Stymne S, Kotapati HK, Sheffield J, Mason C, Bates PD (2019). Specialized lysophosphatidic acid acyltransferases contribute to unusual fatty acid accumulation in exotic *Euphorbiaceae* seed oils. Planta.

[CR41] Shrager J, Hauser C, Chang CW, Harris EH, Davies J, McDermott J, Tamse R, Zhang Z, Grossman AR (2003). *Chlamydomonas reinhardtii* genome project. A guide to the generation and use of the cDNA information. Plant Physiol.

[CR42] Steinert J, Schiml S, Fauser F, Puchta H (2015). Highly efficient heritable plant genome engineering using Cas9 orthologues from *Streptococcus thermophilus* and *Staphylococcus aureus*. Plant J.

[CR43] Tang X, Zheng X, Qi Y, Zhang D, Cheng Y, Tang A, Voytas DF, Zhang Y (2016). A single transcript CRISPR-Cas9 system for efficient genome editing in plants. Mol Plant.

[CR44] Tang X, Lowder LG, Zhang T, Malzahn AA, Zheng X, Voytas DF, Zhong Z, Chen Y, Ren Q, Li Q, Kirkland ER, Zhang Y, Qi Y (2017). A CRISPR-Cpf1 system for efficient genome editing and transcriptional repression in plants. Nat Plants.

[CR45] The Arabidopsis Genome Initiative (2000). Analysis of the genome sequence of the flowering plant *Arabidopsis thaliana*. Nature.

[CR46] van de Loo FJ, Broun P, Turner S, Somerville C (1995). An oleate 12-hydroxylase from *Ricinus communis* L. is a fatty acyl desaturase homolog. Proc Natl Acad Sci USA.

[CR47] Wang ZP, Xing HL, Dong L, Zhang HY, Han CY, Wang XC, Chen QJ (2015). Egg cell-specific promoter-controlled CRISPR/Cas9 efficiently generates homozygous mutants for multiple target genes in Arabidopsis in a single generation. Genome Biol.

[CR48] Wang T, Zhang H, Zhu H (2019). CRISPR technology is revolutionizing the improvement of tomato and other fruit crops. Hortic Res.

[CR49] Wilson LOW, O'Brien AR, Bauer DC (2018). The current state and future of CRISPR-Cas9 gRNA design tools. Front Pharmacol.

[CR50] Wong N, Liu W, Wang X (2015). WU-CRISPR: characteristics of functional guide RNAs for the CRISPR/Cas9 system. Genome Biol.

[CR51] Xie K, Minkenberg B, Yang Y (2015). Boosting CRISPR/Cas9 multiplex editing capability with the endogenous tRNA-processing system. Proc Natl Acad Sci USA.

[CR52] Yan L, Wei S, Wu Y, Hu R, Li H, Yang W, Xie Q (2015). High-efficiency genome editing in *Arabidopsis* using YAO promoter-driven CRISPR/Cas9 system. Mol Plant.

[CR53] Yan F, Kuang Y, Ren B, Wang J, Zhang D, Lin H, Yang B, Zhou X, Zhou H (2018). Highly efficient AT to GC base editing by Cas9n-Guided tRNA adenosine deaminase in rice. Mol Plant.

[CR54] Zabinyakov N, Bullivant G, Cao F, Fernandez Ojeda M, Jia ZP, Wen XY, Dowling JJ, Salomons GS, Mercimek-Andrews S (2017). Characterization of the first knock-out aldh7a1 zebrafish model for pyridoxine-dependent epilepsy using CRISPR-Cas9 technology. PLoS ONE.

[CR56] Zhang Li Z, Li JF (2016). Targeted gene manipulation in plants using the CRISPR/Cas technology. J Genet Genom.

[CR55] Zhang Q, Xing HL, Wang ZP, Zhang HY, Yang F, Wang XC, Chen QJ (2018). Potential high-frequency off-target mutagenesis induced by CRISPR/Cas9 in *Arabidopsis* and its prevention. Plant Mol Biol.

[CR57] Zong Y, Wang Y, Li C, Zhang R, Chen K, Ran Y, Qiu J, Wang D, Gao C (2017). Precise base editing in rice, wheat and maize with a Cas9-cytidine deaminase fusion. Nat Biotechnol.

