# Supplementary material for: Gene editing in plants: assessing the variables through a simplified case study
Source: Plant Mol Biol. 2020 Feb 10;103(1):75–89. doi: 10.1007/s11103-020-00976-2 (PMC7170989; doi:10.1007/s11103-020-00976-2)
Supplement: Supplementary file 1 — Supplementary material 1 Supplementary Fig. 1 Examples of specific types of Cas9-mediated mutagenesis in vegetative tissues of Arabidopsis E113. Genomic DNA was isolated from fluorescence-depleted T1 leaf tissue of E640 and E642 plants, the DsRed ORF amplified by PCR, and digested with NcoI or PstI as appropriate. Restriction-resistant amplicons were purified, cloned, and sequenced. Representative examples are shown below, aligned to the native DsRed sequence. Query = native DsRed. Subject = cloned sequence from E640/E642 CRISPR plants (DOCX 24 kb) [file 11103_2020_976_MOESM1_ESM.docx]

**E642 (AG of underlined *Pst*I site = first two bases of PAM)**

Query 301 GACGGCGGCGTGGCGACCGTGACCCAGGACTCCTCC**CTGCAG**GACGGCTGCTTCATCTAC 360

|||||| ||||| |||||||||||||||||||||| |||

Sbjct 484 GACGGCCGCGTGTCGACCGTGACCCAGGACTCCTC----------------------TAC 447

Query 301 GACGGCGGCGTGGCGACCGTGACCCAGGACTCCTCC**C-TGCAG**GACGGCTGCTTCATCTA 359

||||||||||||||||||||||||||||||||||||| ||||||||||||||||||||||

Sbjct 391 GACGGCGGCGTGGCGACCGTGACCCAGGACTCCTCCCTTGCAGGACGGCTGCTTCATCTA 450

Query 301 GACGGCGGCGTGGCGACCGTGACCCAGGACTCCTCC**CTGCAG**GACGGCTGCTTCATCTAC 360

||||||||||||||||||||||||||||||||||||| ||||||||||||||||||||||

Sbjct 391 GACGGCGGCGTGGCGACCGTGACCCAGGACTCCTCCC-GCAGGACGGCTGCTTCATCTAC 449

**E640 (TGG of underlined *Nco*I site is PAM site)**

Query 301 CCCCTCCGACGGCCCCGTGATGCAGAAGAAGA**CCATGG**GCTGGGAGGCCTCCACCGAGCG 360

||||||||||||||||||||||||||||||| ||||||||||||||||||||||||||

Sbjct 482 CCCCTCCGACGGCCCCGTGATGCAGAAGAAG---ATGGGCTGGGAGGCCTCCACCGAGCG 538

Query 301 CCCCTCCGACGGCCCCGTGATGCAGAAGAAGA**CCATGG**GCTGGGAGGCCTCCACCGAGCG 360

|||||||||||||||||||| ||||||||||||||||||||

Sbjct 477 CCCCTCCGACGGCCCCGTGA--------------------TGGGAGGCCTCCACCGAGCG 516

Supplementary Fig. 1 Examples of specific types of Cas9-mediated mutagenesis in vegetative tissues of *Arabidopsis* E113. Genomic DNA was isolated from fluorescence-depleted T_1_ leaf tissue of E640 and E642 plants, the *DsRed* ORF amplified by PCR, and digested with *Nco*I or *Pst*I as appropriate. Restriction-resistant amplicons were purified, cloned, and sequenced. Representative examples are shown below, aligned to the native *DsRed* sequence. Query = native *DsRed*. Subject = cloned sequence from E640/E642 CRISPR plants
