# Supplementary material for: Gene editing in plants: assessing the variables through a simplified case study
Source: Plant Mol Biol. 2020 Feb 10;103(1):75–89. doi: 10.1007/s11103-020-00976-2 (PMC7170989; doi:10.1007/s11103-020-00976-2)
Supplement: Supplementary file 4 — Supplementary material 4 Supplementary Fig. 4 Mutant FAH12 loci CRISPR CL37 plants display typical types of Cas9-generated mutations. Cas9-mutated FAH12 PCR amplicons were produced by PCR, from genomic DNA isolated from E719 (UBQ10p:sgRNAAtACT8p:Cas9) and E720 (UBQ10p:sgRNA + YAOp:Cas9) plants that contained reduced amounts of seed HFA. After treatment with BstXI, restriction-resistant PCR products were purified, cloned, and sequenced. Seventeen copies were analyzed, which included: 9 single bp insertions, 3 single-bp deletions, two 4-bp deletions, one 6-bp deletion, one 7-bp deletion, and one 12-bp deletion. Representative sequences are shown below (subject), aligned to native FAH12 sequence (query). The PAM333 sequence is underlined, the recognition sequence for BstXI used to screen for mutations is boxed in yellow. See Fig. 2 for more details (DOCX 15 kb) [file 11103_2020_976_MOESM4_ESM.docx]

Supplementary Fig. 4 Mutant *FAH12* loci CRISPR CL37 plants display typical types of Cas9-generated mutations. Cas9-mutated *FAH12* PCR amplicons were produced by PCR, from genomic DNA isolated from E719 (*UBQ10p:sgRNA* + *AtACT8p:Cas9*) and E720 (*UBQ10p:sgRNA* + *YAOp:Cas9)* plants that contained reduced amounts of seed HFA. After treatment with *BstX*I, restriction-resistant PCR products were purified, cloned, and sequenced. Seventeen copies were analyzed, which included: 9 single bp insertions, 3 single-bp deletions, two 4-bp deletions, one 6-bp deletion, one 7-bp deletion, and one 12-bp deletion. Representative sequences are shown below (subject), aligned to native *FAH12* sequence (query). The PAM333 sequence is underlined, the recognition sequence for *BstX*I used to screen for mutations is boxed in yellow. See Fig. 2 for more details

E719-1 #1

Query 301 ATTCTCACTGGTCTTTGGGTCATCGGCCATGAATGTGGCCATCATGCTTTTAGTGAGTAT 360

Sbjct 336 ATTCTCACTGGTCTTTGGGTCATCGGCCATG-ATGTGGCCATCATGCTTTTAGTGAGTAT 394

E719-1 #4

Query 301 ATTCTCACTGGTCTTTGGGTCATCGGCCATGAATGTGGCCATCATGCTTTTAGTGAGTAT 360

Sbjct 335 ATTCTCACTGGTCTTTGGGTCATCGGCC----ATGTGGCCATCATGCTTTTAGTGAGTAT 390

E719-2 #6

Query 301 ATTCTCACTGGTCTTTGGGTCATCGGCCATGAATGTGGCCATCATGCTTTTAGTGAGTAT 360

Sbjct 338 ATTCTCACTGGTCTTTGGGT------------ATGTGGCCATCATGCTTTTAGTGAGTAT 385

E720-1 #3

Query 301 ATTCTCACTGGTCTTTGGGTCATCGGCCATGAATGTGGCCATCATGCTTTTAGTGAGTAT 360

Sbjct 336 ATTCTCACTGGTCTTTGGGTCATCGGCCATGA-------CATCATGCTTTTAGGGAGTAT 388

E720-1 #4

Query 301 ATTCTCACTGGTCTTTGGGTCATCGGCCATG-AATGTGGCCATCATGCTTTTAGTGAGTA 359

Sbjct 332 ATTCTCACTGGTCTTTGGGTCATCGGCCATGAAATGTGGCCATCATGCTTTTAGTGAGTA 391
