# Supplementary material for: Gene editing in plants: assessing the variables through a simplified case study
Source: Plant Mol Biol. 2020 Feb 10;103(1):75–89. doi: 10.1007/s11103-020-00976-2 (PMC7170989; doi:10.1007/s11103-020-00976-2)
Supplement: Supplementary file 7 — Supplementary material 7 (DOCX 13 kb) [file 11103_2020_976_MOESM7_ESM.docx]

Supplementary Table 1 Nucleotide sequence of *RcFAH12* ORF expressed in *A. thaliana* CL37. PAMs located at nucleotide positions 333, 540, and 885 are boxed, and the respective protospacers are underlined. See Fig. 3 for additional details

| RcFAH12  ATGGGAGGTGGTGGTCGCATGTCTACTGTCATAACCAGCAACAACAGTGAGAAGAAAGGAGGAAGCAGCCACCTTAAGCGAGCGCCGCACACGAAGCCTCCTTTCACACTTGGTGACCTCAAGAGAGCCATCCCACCCCATTGCTTTGAACGCTCTTTTGTGCGCTCATTCTCCTATGTTGCCTATGATGTCTGCTTAAGTTTTCTTTTCTACTCGATCGCCACCAACTTCTTCCCTTACATCTCTTCTCCGCTCTCGTATGTCGCTTGGCTGGTTTACTGGCTCTTCCAAGGCTGCATTCTCACTGGTCTTTGGGTCATCGGCCATGAATGTGGCCATCATGCTTTTAGTGAGTATCAGCTGGCTGATGACATTGTTGGCCTAATTGTCCATTCTGCACTTCTGGTTCCATATTTTTCATGGAAATATAGCCATCGCCGCCACCATTCTAACATAGGATCTCTCGAGCGAGACGAAGTGTTCGTCCCGAAATCAAAGTCGAAAATTTCATGGTATTCTAAGTACTTAAACAACCCGCCAGGTCGAGTTTTGACACTTGCTGCCACGCTCCTCCTTGGCTGGCCTTTATACTTAGCTTTCAATGTCTCTGGTAGACCTTACGATCGCTTTGCTTGCCATTATGATCCCTATGGCCCAATATTTTCCGAAAGAGAAAGGCTTCAGATTTACATTGCTGACCTCGGAATCTTTGCCACAACGTTTGTGCTTTATCAGGCTACAATGGCAAAAGGGTTGGCTTGGGTAATGCGTATCTATGGGGTGCCATTGCTTATTGTTAACTGTTTCCTTGTTATGATCACATACTTGCAGCACACTCACCCAGCTATTCCACGCTATGGCTCATCGGAATGGGATTGGCTCCGGGGAGCAATGGTGACTGTCGATAGAGATTATGGGGTGTTGAATAAAGTATTCCATAACATTGCAGACACTCATGTAGCTCATCATCTCTTTGCTACAGTGCCACATTACCATGCAATGGAGGCCACTAAAGCAATCAAGCCTATAATGGGTGAGTATTACCGGTATGATGGTACCCCATTTTACAAGGCATTGTGGAGGGAGGCAAAGGAGTGCTTGTTCGTCGAGCCAGATGAAGGAGCTCCTACACAAGGCGTTTTCTGGTACCGGAACAAGTATTAA |
| --- |
