# Supplementary material for: Gene editing in plants: assessing the variables through a simplified case study
Source: Plant Mol Biol. 2020 Feb 10;103(1):75–89. doi: 10.1007/s11103-020-00976-2 (PMC7170989; doi:10.1007/s11103-020-00976-2)
Supplement: Supplementary file 8 — Supplementary material 8 (DOCX 14 kb) [file 11103_2020_976_MOESM8_ESM.docx]

Supplementary Table 2 Nucleotide sequences of promoters used to express sgRNA and Cas9 components of plasmid constructs shown in Fig. 2. All promoters were purchased commercially. Synthetic copies of the promoters were cloned and sequenced to confirm accuracy prior to use in sgRNA and Cas9 plasmid construction. See original references (*AtACT8p*, An et al. 1996; *EC1.2en-EC1.1p*, Wang et al. 2015; *YAOp*, Yan et al. 2015; *AtUBQ10p*, Zhang et al. 2016) for additional information

| *AtACT8p*  AAGCCCAAATTTCTCTAAAATTTACATACTTCTATCTAATAATAGAGTTACATACTTCTATCTAAAAAGTATAATAGAGTCACATACTTCTATCTAATATGTAACAATATAACATAACACGTAATTTGTTTTATTATGAAATAAAAATCATGTAATTATAAAATAAAAATACATGTGATAAAATTGTCTAGTGATTAATCAATGGTTCCTACACAATGTTCTAAATATTTTAGTAAATTTTACTAGCTAATAGATGGAAACTTATCGCATGTTACAGGAGTAGTTCATCGTGGCCTTAGTAAGTTATTGATAAAGTTGTCCATTTTATGTGTTGTTGTCAAATTGTTTTTGTTTTTTGTATTTTTTTTTGAATAAGTTGTTATCAAATTATAATGTCTAATACTACTATAGAAATTTATCACTTTTATCCTCTCTTTTTGTTATGTCTTTTTCCTTTCAAAATTGCATACCATTTTGTATTCTTTTCTCACCAAACTTATTCAAACTAAATTTCCAAACATATTATAGAGAACTATCAAAATACAAATAGTTACATAAACAACATAAGTACAAACAAAATCACGAGAAAAAGTGAAATTATATTACAAAATGCTATATTTTTTTCTCACACTCTATATTAATGTCAAATATGAGTAATTTCAATCAAAAGCCATTTTTCTTTTGCATAATTCATGTTTATTTTTTTATTTTTTTCATCTTGCATAATTCATGTTTAAAAGGATATATACATGGGTCTACTACATTCACCTGACATTACGTTTTATGTGTTTGTCTTCTGAAAATAATCATCAAAATATTTCAGGACTTGTTTACGTTTTCAGGAGAAAAAAAATAACTGTACCCTTTTCAATATAGAAATAACATTTGTAGAAATCGTGGATTTTCCTTAATAAACAATCCAAAACACGACCACCGTTGTCTCCTCGACTCGGTAACACCCGATCGCCGACTTGAAAATTAGAAGAAAAATGAAAAGAATAATAAAAAAAAAAAAGGAATGATTATTGAAGCTGTCATATATGTCGACCCTATCACAGTCAATCCAATAGCCTATATTCGCCAACTGATATATCCAACGGCTCACAAATTTTCACAAACTTTTCAAAAAAGTATAAATAAAAGAGGCTGTCTGACAGCCATGTCACGTTATACTTTTTCCGTATGATCGAAATGATTCGTCTTTGTCGAATTTAATTATTTCCAAAATTGATGACTCTAAAGAAAAAAAAATAGTTTTTCAGATAAACCCGCCTATATAAATAGTTCAACACTCGGTTTATTTCTTCTCCCCTCTTTGAATTGCCTCGTCGTCTTCAGCTTCATCGGCCGTTGCATTTCCCGGCGATAAGAGAGAGAAAGAGGAGAAAGAGTGAGCCAGATCTTCATCGTCGTGGTTCTTGTTTCTTCCTCGATCTCTCGATCTTCTGCTTTTGCTTTTCCGATTAAG |
| --- |
| *EC1.2en-EC1.1p*  GAATAAAAGCATTTGCGTTTGGTTTATCATTGCGTTTATACAAGGACAGAGATCCACTGAGCTGGAATAGCTTAAAACCATTATCAGAACAAAATAAACCATTTTTTGTTAAGAATCAGAGCATAGTAAACAACAGAAACAACCTAAGAGAGGTAACTTGTCCAAGAAGATAGCTAATTATATCTATTTTATAAAAGTTATCATAGTTTGTAAGTCACAAAAGATGCAAATAACAGAGAAACTAGGAGACTTGAGAATATACATTCTTGTATATTTGTATTCGAGATTGTGAAAATTTGACCATAAGTTTAAATTCTTAAAAAGATATATCTGATCTAGGTGATGGTTATAGACTGTAATTTTACCACATGTTTAATGATGGATAGTGACACACATGACACATCGACAACACTATAGCATCTTATTTAGATTACAACATGAAATTTTTCTGTAATACATGTCTTTGTACATAATTTAAAAGTAATTCCTAAGAAATATATTTATACAAGGAGTTTAAAGAAAACATAGCATAAAGTTCAATGAGTAGTAAAAACCATATACAGTATATAGCATAAAGTTCAATGAGTTTATTACAAAAGCATTGGTTCACTTTCTGTAACACGACGTTAAACCTTCGTCTCCAATAGGAGCGCTACTGATTCAACATGCCAATATATACTAAATACGTTTCTACAGTCAAATGCTTTAACGTTTCATGATTAAGTGACTATTTACCGTCAATCCTTTCCCATTCCTCCCACTAATCCAACTTTTTAATTACTCTTAAATCACCACTAAGCTAACGCCTATCATGAATTAGCTCTACTAAATCTAGCAACCTTTCAAATTTGCAGTATTGCAGGTGTCTCTGTGTCTTTAAAATAGTTGCCTTATGATTTCTTCGGTTTCAAGATGATCAAATAGTTATAGATTTCATGCTCACACATGCTCATTAGATGTGTACATACTTTACTTACCCAAATCTATTTTCTCGCAAAGATTTTGATGGTAAAGCTGATTTGGTTCTATTGAACTAAATCAAACGAGTTTCAGACTGAGTGATTCTAATCCGGCCCATTAGCCCCTAAACAGACCCACTAATTACGCAGCTTTTAATAGAGTAATTACACCTAGTTTACCCACTAAACCACTAAGCACTAATTATCTCACAATCTAATGAGCTTCCCTCGTAATTACTTGGGCTTTCACTCTACCATTTATTTGTAACAGTCAAGTCTCTACTGTCTCTATATAAACTCTCTAAAGTTAACACACAATTCTCATCACAAACAAATCAACCAAAGCAACTTCTACTCTTTCTTCTTTCGACCTTATCAATCTGTTGAGAA |
| *YAOp*  GATGGGAAATTCATTGAAAACCCTAAACCCAAATCAACAGCTGCAATTCAAAAGGGGACTAATTGACAAACAAAAATTGATAACAAATAGAGGTAGGGGGAGAGTTTCGTACGCGACAATGAGATTGAGCTCTTGAGGACTTGTGAAGTTGCCAACGCACGAGTGAGTGACACTGGTCGGTTTGTGAGCCGTAACAACGTAGTTCCATGAGCTCATCTTCCTCTTCTTTGTCTCCAGGGAATTTGAGTTCGACTTTCTACGCGAGGGCCCTCGAGGAAGCTTCTAGATTTCTGAATCGAGCTTTCGGAATTTTAACATAGAGAAGTTAGAGAGAGAATGAAAAGCCAAAGGAGGCGAAAATCGAACAAGGAAGAAGAAAGACAACTTTCGACAAAGACTGGTCGGTCGGTTTTGGTAGACAATTGAAATTAGATGGATGGTCCGGTTCGGTATACTATAAGATTAAAAACAGTTTTAAATTCAGCTAAACCGAACTCATTTGATTTTATTAAACCGGAATCATCCGATTCGAGTTTGTAAAAAATACCGAAATTGAAAACACTAAACAAAAACTGTATTAAACTGTTACTGAAATAAGAGAATCTCCCAATTCGGTTTACGTACTACTCTTCAGAAATCAGAACCAAAAATTCAGAAATCGGATTGAACCAAACTTAAATTGACGGTCCGGTTAGTCTTCGGCTCTACAAATTAAAGGCCCAAGTTTCTGCTTTAAAAGAACGAAATAGTTAATGGGCTCAAACCATAGACCAGGTAAGTCATGGGCTTGGTTAGTCCGGGTCAACCCGGTAGACCCGATTCCTGAAGAAAACCTAGTGGAAGGTTTAAAGTTGTAAACTTTCCGACCAAATAAACAAAATCGTTTTCCAGCTTCTTCCGTCGCCACTAAACCCTGAGGCTAAACCTAGACGAGTCAAAGTGTAAAATCGTTAAACCCTAAGAGGGAGTGAGAGAGAGAAGA |
| *AtUBQ10p*  CGACGAGTCAGTAATAAACGGCGTCAAAGTGGTTGCAGCCGGCACACACGAGTCGTGTTTATCAACTCAAAGCACAAATACTTTTCCTCAACCTAAAAATAAGGCAATTAGCCAAAAACAACTTTGCGTGTAAACAACGCTCAATACACGTGTCATTTTATTATTAGCTATTGCTTCACCGCCTTAGCTTTCTCGTGACCTAGTCGTCCTCGTCTTTTCTTCTTCTTCTTCTATAAAACAATACCCAAAGAGCTCTTCTTCTTCACAATTCAGATTTCAATTTCTCAAAATCTTAAAAACTTTCTCTCAATTCTCTCTACCGTGATCAAGGTAAATTTCTGTGTTCCTTATTCTCTCAAAATCTTCGATTTTGTTTTCGTTCGATCCCAATTTCGTATATGTTCTTTGGTTTAGATTCTGTTAATCTTAGATCGAAGACGATTTTCTGGGTTTGATCGTTAGATATCATCTTAATTCTCGATTAGGGTTTCATAGATATCATCCGATTTGTTCAAATAATTTGAGTTTTGTCGAATAATTACTCTTCGATTTGTGATTTCTATCTAGATCTGGTGTTAGTTTCTAGTTTGTGCGATCGAATTTGTCGATTAATCTGAGTTTTTCTGATTAACA |
